# Red Tape or Phthisiophobia?

**Published:** 1913-07

**Authors:** 


### Red Tape or Phthisiophobia?

The *Buffalo Evening News* has recently called attention to the case of a girl dying of tuberculosis and endangering three other children compelled by poverty to share the same room with her. No hospital could be found to take the case, under existing conditions. What impresses us particularly is that the means suggested for solving the problem are the erection of a temporary tuberculosis hospital and, in the emergency, erecting a tent to accommodate the child at an out-door camp.

We do not, by any means, disapprove the general opinion that tuberculosis, by virtue of its prevalence and certain characteristic features, is best treated at special institutions nor do we ignore the danger of the bacillus. In fact, our friend S. A. Knopf has called us a phthisiophobe because we do not subscribe to the recent dictum that the cleanly consumptive may be cared for in his own home without danger. But there are plain humanitarian duties which ought to outrank the more or less theoretic duty of considering the interests of the uninfected, in cases of this degree.

It seems only a short time since the writer was regarded as very young and germ-crazy for protesting against the treatment of consumptives in public wards along with a dozen or more patients of other kinds. The pendulum of professional opinion has certainly swung very fast if it is now considered impossible to give the tuberculous poor, housing and nursing and relief in a hopeless case, in a general hospital for fear of infecting other inmates. Without reviewing details of conveyance of infection, etc., familiar to all students, we feel justified in claiming that there is no hospital of modern equipment which could not treat consumptives in a separate ward with such precautions as to render transmission of the disease practically impossible. And, in an advanced case, beyond the stage at which climate, fresh air, and other hygienic means are necessary because they add favorable factors to the treatment of a case in which hope of cure remains, we can see no reason why a special hospital is at all essential, however desirable it may be for various reasons.

### Three Kinds of Tuberculous Hospitals.

For the sake of facilitating criticism and of making the matter plain, especially since the care of the tuberculous has, at last, enlisted popular enthusiasm and arguments have become crystalized into slogans whose full meaning is not apparent to all, we may be pardoned for presenting some thoughts in a rather dogmatic way.

1. The provision of special hospitals for tuberculous patients is not nearly so important as the provision of still more highly specialized institutions for different types of tuberculosis.

2. From the practical standpoint of prophylaxis and therapeutics, tuberculosis resolves itself into three principal groups: I. Latent and susceptible (according to the lay nomenclature, "threatened") cases; II. Moderately advanced, but still relatively hopeful cases, with discharge of bacilli, so that, with appropriate qualifications and apologies, they may be termed "contagious"; III. Far advanced, hopeless cases which are "contagious" not only in the sense applicable to the preceding group but because the prostration and mental hebetude or actual unconsciousness or mental perturbation of the patient prevents him from practicing the precautions which minimize the danger of "contagion" in conscientious and intelligent patients of the second group. It is self-evident that these lines cannot always be drawn with exactness in the practical assortment of cases and that they do not complete either the pathologic or the prophylactic and therapeutic grouping, especially with regard to non-pulmonary cases. They suffice, however, for present purposes.

3. It is obvious that, merely from the psychic standpoint, it is desirable to seggregate these groups from one another, not only for the comfort of the patients but to secure the ultimate results aimed at.

4. Even the economic maintenance of institutions requires the sharp division indicated and the word economic should be understood as applying to the individual as well as to the supporting community. The first group of patients require very little technical nursing, comparatively little individualized dieting, much fresh air but probably no special climate, moderate exercise and, consisting largely of young persons, considerable direction and discipline, not only in the ordinary sense but in that of imitating, so far as practical, the ordinary routine, educational and otherwise, of normal young persons. To mix such a group of individuals with more advanced patients requiring formal nursing, individual diet, almost complete rest, and what may be termed quarantine measures against mutual infection and establishment of fomites, quite aside from the actual danger to the incipient group, requires a much more expensive plant and higher maintenance charges. Again, the incipient group is not usually regarded as requiring any special climate, provided the location of the institution is salubrious, whereas the question of climate for the second group is sub judice. The third group, again, being mostly bed-ridden and, barring very desirable errors in prognosis, doomed to death in a comparatively short time, requires quite different details of institutional management from the second. There is the same physic objection to mixing the two groups, climate is a negligible factor and propinquity to the home of the patient is far more important than in the first two groups. It would be going too far to say that the management of the dependent tuberculous class or of tuberculous patients in general as a sociologic problem, should not be unified so far as possible and there are circumstances such as small population of the political unit concerned, or fewness of its tuberculous cases, or the coincidence of eligible sites in the immediate neighborhood, which will justify the treatment of all groups of cases in the same institution, with such subdivision and seggregation as may conform to the general principles stated.

